# HLA-A, -B, -C and -DRB1 Association with Autism Spectrum Disorder Risk: A Sex-Related Analysis in Italian ASD Children and Their Siblings

**DOI:** 10.3390/ijms25189879

**Published:** 2024-09-12

**Authors:** Franca Rosa Guerini, Elisabetta Bolognesi, Martina Maria Mensi, Michela Zanette, Cristina Agliardi, Milena Zanzottera, Matteo Chiappedi, Silvia Annunziata, Francisco García-García, Anna Cavallini, Mario Clerici

**Affiliations:** 1 Laboratory of Molecular Medicine and Biotechnologies, IRCCS Fondazione Don Carlo Gnocchi, Via Capecelatro 66, 20148 Milan, Italy; fguerini@dongnocchi.it (F.R.G.); mzanette@dongnocchi.it (M.Z.); cagliardi@dongnocchi.it (C.A.); mzanzottera@dongnocchi.it (M.Z.); sannunziata@dongnocchi.it (S.A.); ancavallini@dongnocchi.it (A.C.); mario.clerici@unimi.it (M.C.); 2 Department of Brain and Behavioural Sciences, University of Pavia, 27100 Pavia, Italy; martina.mensi@mondino.it; 3 IRCCS Fondazione Mondino, 27100 Pavia, Italy; 4 Child Neurology and Psychiatry Unit, ASST Pavia, 27029 Vigevano, Italy; 5 Computational Biomedicine Laboratory, Principe Felipe Research Center (CIPF), C/Eduardo Primo Yúfera 3, 46012 Valencia, Spain; fgarcia@cipf.es; 6 Pathophysiology and Transplantation Department, University of Milan, 20122 Milan, Italy

**Keywords:** autism spectrum disorders, human leukocyte antigen (HLA), sex-bias disease, immunogenetics, sex-based risk factors

## Abstract

Autism Spectrum disorders (ASD) are diagnosed more often in males than in females, by a ratio of about 3:1; this is likely to be due to a difference in risk burden between the sexes and/or to “compensatory skills” in females, that may delay the diagnosis of ASD. Identifying specific risk factors for ASD in females may be important in facilitating early diagnosis. We investigated whether HLA- class I: -A, -B, -C and class II -DRB1 alleles, which have been suggested to play a role in the development of ASD, can be considered as sex-related risk/protective markers towards the ASD. We performed HLA allele genotyping in 178 Italian children with ASD, 94 healthy siblings, and their parents. HLA allele distribution was compared between children with ASD, sex-matched healthy siblings, and a cohort of healthy controls (HC) enrolled in the Italian bone marrow donor registry. Allele transmission from parents to children with ASD and their siblings was also assessed. Our findings suggest that HLA-A*02, B*38, and C*12 alleles are more frequently carried by females with ASD compared to both HC and healthy female siblings, indicating these alleles as potential risk factors for ASD in females. Conversely, the HLA-A*03 allele was more commonly transmitted to healthy female siblings, suggesting it might have a protective effect. Additionally, the HLA-B*44 allele was found to be more prevalent in boys with ASD, indicating it is a potential risk factor for male patients. This is the first Italian study of sex-related HLA association with ASD. If confirmed, these results could facilitate early ASD diagnosis in female patients, allowing earlier interventions, which are crucial in the management of neurodevelopmental disorders.

## 1. Introduction

Autism spectrum disorders (ASDs) are neurodevelopmental dysfunctions characterized by difficulties in social interactions and a tendency toward repetitive and/or stereotyped behaviors. Today, ASDs affect approximately 1 in 59 individuals worldwide, with a male-to-female ratio of approximately 3:1 [1]. Specific behavioral differences are known to occur in female and male children with ASD. Thus, male children with ASD tend to have elevated language and motor scores and are more likely to be hyperactive, aggressive, and impulsive [2,3,4,5]. Females, on the other hand, perform better in executive function, visual reception, and attention to faces, but they are more prone to develop depression, anxiety, and/or other emotional symptoms in adolescence [2,4]. The putative compensatory ability in females helps them in social interaction contexts [6] and makes clinical diagnosis more difficult in childhood. For these reasons, the diagnosis of ASD in girls may be underestimated, often resulting in the impossibility of initiating early behavioral recovery interventions. It is, therefore, of utmost importance to identify specific risk factors for ASD in girls to facilitate early diagnosis.

The most accepted theory of ASD-biased male prevalence is based on the hypothesis that specific factors may protect females from developing ASD [7]. This is consistent with the idea that a greater genetic/mutational load is needed for females to reach a formal diagnosis of ASD. It has also been suggested that a greater aetiological burden appears to be required for behavioral impairment to manifest in females, possibly due to genetic and hormonal factors [8]. Recent studies examining sex differences in gene expression between brain regions suggested that functionally different gene families are preferentially activated in female and male brains. Neuron-specific transcripts were increased in male brains, whereas transcripts related to immune and glial function were more abundant in female brains [9,10,11,12].

The immune hypothesis is considered to be a major factor contributing to the pathogenesis of ASD, as well as a way to explain the differences in ASD clinical phenotypes and comorbidities that influence disease course and severity. Several studies support the association of different genes involved in the immune system with the development of ASD [13]. Among the immune genes, the Major Histocompatibility Complex (MHC) region has been widely implicated in conferring an increased risk of ASD [14,15,16]. MHC is a highly polymorphic cluster of genes with some of the greatest allelic diversity in the genome that mediate both adaptive and innate immune responses [17,18,19]. This region contains a cluster of genes that play a prominent role in regulating immuno-inflammatory processes, as well as in neurodevelopment and neuroplasticity, through microglial regulation and synaptic pruning [20]. Converging evidence shows that HLA class I and class II alleles play a relevant role in ASD, as well as in schizophrenia and bipolar disorder [21], strongly suggesting the role of HLA alleles in early development and the course of psychiatric disorders.

Although GWAS studies suggest that the HLA region modulates the ASD risk [15,16], candidate gene analysis of potential associations between HLA genetic diversity and ASD has yielded controversial results [22]. Investigation of the relationship between HLA and ASD in different clinical profiles and sexes has been recommended.

We investigated whether a risk and/or protective role could be attributed to any HLA allele selectively to females or males. To this end, the distribution of the most studied classic HLA class I: HLA -A, -B, -C, and class II HLA-DRB1 alleles were analyzed in 178 Italian children with ASD and compared with that of a group of Italian healthy controls [23] and of sex-matched healthy siblings (SIBS).

## 2. Results

**Figure 2:**
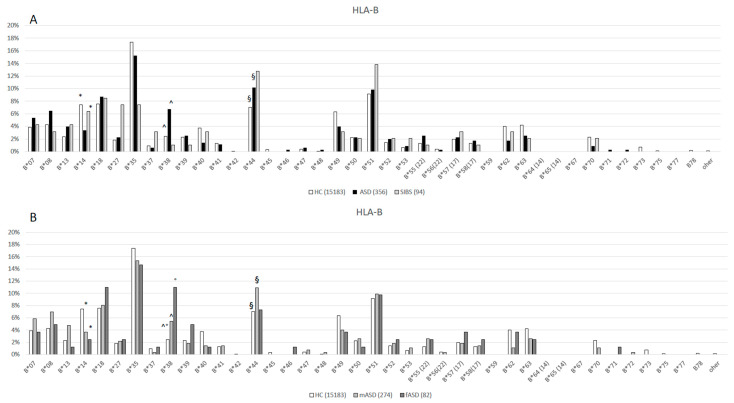

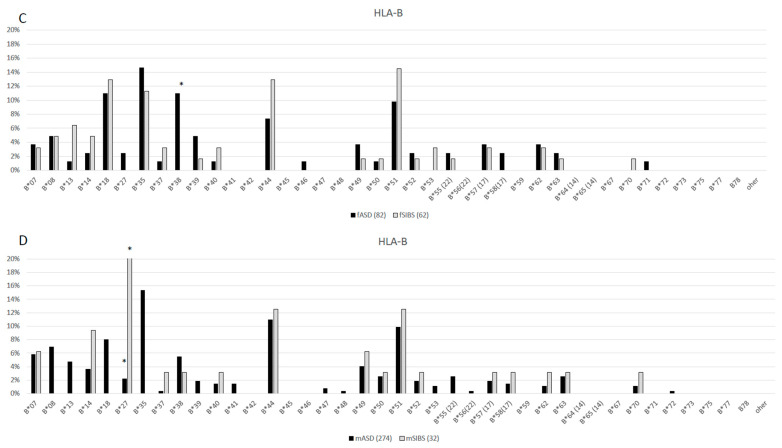
(**A**): HLA-B allele distribution in 7591 HC [23], 178 ASD children (ASD vs. HC *p_c_* < 0.001, df = 36) and 47 healthy SIBS (ASD vs. SIBS *p_c_* = 0.29, df = 29). * (*p* = 0.002, *p_c_*= 0.07 OR: 0.4; 95%CI: 0.2–0.8); ^ (*p* < 0.0001, *p_c_* < 0.001 OR: 2.9; 95%CI: 1.9–4.4); § (*p* = 0.03, *p_c_*=0.72 OR: 1.5; 95%CI: 1.1–2.1) (**B**): HLA-B allele distribution in 7591 HC [23], 41 female ASD (fASD) (fASD vs. HC *p_c_* < 0.001, df=35), and 137 male ASD (mASD) (mASD vs. HC *p_c_*< 0.001, df = 34) * (*p* = 0.01, *p_c_* = 0.36 OR: 0.5; 95%CI: 0.2–0.8) ° (*p* < 0.001, *p_c_* = 0.01 OR: 4.9; 95%CI: 2.2–3.4); ^ (*p* = 0.006, *p_c_* = 0.21 OR: 2.4; 95%CI: 1.3–3.9); § (*p* = 0.02, *p_c_* = 0.70 OR: 1.6; 95%CI: 1.1–2.4) (**C**): HLA-B allele distribution in 41 female ASD (fASD) and 31 female sibs (fSIBS) (fASD vs. fSIBS *p_c_* = 0.44, df = 24) * (*p_f_* = 0.01, *p_c_* = 0.24 df = 24); (**D**): HLA-B allele distribution in 137 male ASD (mASD) and 16 male sibs (mSIBS) (mASD vs. mSIBS *p_c_* = 0.005, df = 27) * (*p* = 0.0001, *p_c_* = 0.003 OR: 0.08; 95%CI: 0.02–0.3).

**Figure 3:**
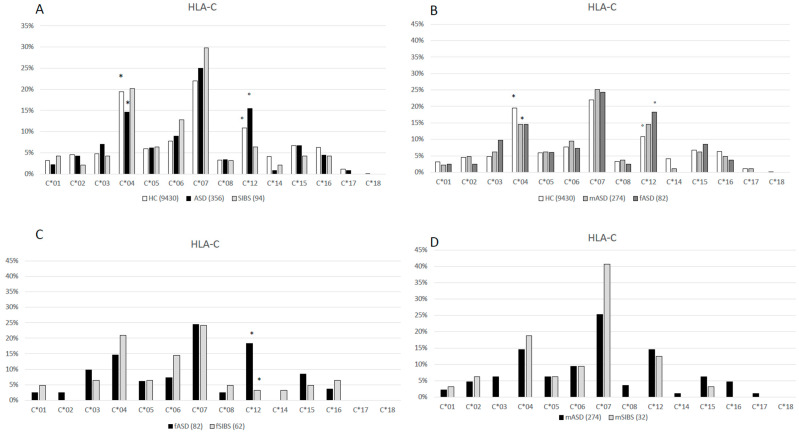
(**A**): HLA-C allele distribution in 4715 HC [23], 178 ASD children (ASD vs. HC *p_c_* = 0.006, df = 13) and 47 healthy SIBS (ASD vs. SIBS *p_c_* = 0.35, df = 12). * (*p* = 0.02, *p_c_* = 0.26 OR: 0.7; 95%CI: 0.5–0.9); ° (*p* = 0.01, *p_c_* = 0.13 OR: 1.5; 95%CI: 1.1–2.0). (**B**): HLA-C allele distribution in 4715 HC [23], 41 female ASD (fASD) (fASD vs. HC *p_c_* = 0.23, df = 13), and 137 male ASD (mASD) (mASD vs. HC *p_c_* = 0.14, df = 13); * (*p* = 0.04, *p_c_* = 0.52 OR: 0.7; 95%CI: 0.5–1.0); ° (*p* = 0.05, *p_c_* = 0.65 OR: 1.8; 95%CI: 1.0–3.1). (**C**): HLA-C allele distribution in 41 fASD and 31 female sibs (fSIBS) (fASD vs. fSIBS *p_c_* = 0.12, df = 11) * (p_f_ = 0.008, *p_c_* = 0.09 OR: 6.6; 95%CI: 1.6–44.5). (**D)**: HLA-C allele distribution in 137 mASD and 16 male sibs (mSIBS) (mASD vs. mSIBS *p_c_* = 0.69, df = 12).

**Figure 4:**
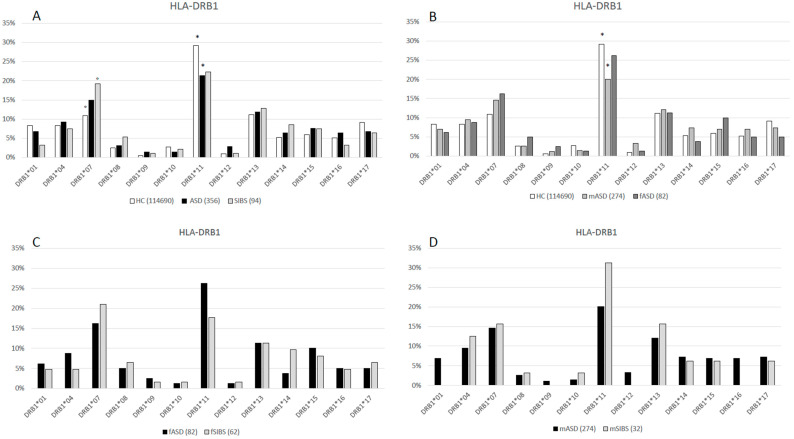
(**A**): HLA-DRB1 allele distribution in 57,345 HC [23], 178 ASD children (ASD vs. HC *p_c_* < 0.001, df = 12), and 47 healthy SIBS (ASD vs. SIBS *p_c_* = 0.86, df = 12) * (*p* = 0.001, *p_c_* = 0.01 OR: 0.7; 95%CI: 0.4–0.8); ° (*p* = 0.02, *p_c_* = 0.24 OR: 1.4; 95%CI: 1.1–1.9). (**B**): HLA-DRB1 allele distribution in 57,345 HC [23], 41 female ASD (fASD) (fASD vs. HC *p_c_* = 0.23, df = 12), and 137 male ASD (mASD) (mASD vs. HC *p_c_* = 0.002, df = 12) * *p* = 0.001 *p_c_* = 0.01 OR: 0.6; 95%CI: 0.5–0.8. (**C**): HLA-DRB1 allele distribution in 41 fASD and 31 female sibs (fSIBS) (fASD vs. fSIBS *p_c_* = 0.96, df = 12). (**D**): HLA-DRB1 allele distribution in 137 mASD and 16 male sibs (mSIBS) (mASD vs. mSIBS *p_c_* = 0.72, df = 12).

**Figure 1:**
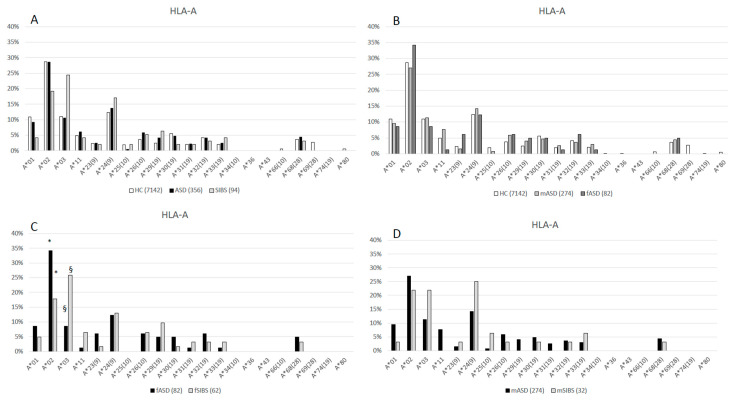
(**A**): HLA-A allele distribution in 3571 HC [23], 178 ASD children (ASD vs. HC *p_c_* = 0.05, df = 19), and 47 healthy SIBS (ASD vs. SIBS *p_c_* = 0.06, df = 13). (**B**): HLA-A allele distribution in 3571 HC [23], 41 female ASD (fASD) (HC vs. fASD *p_c_* = 0.05, df = 19), and 137 male ASD (mASD) (HC vs. mASD *p_c_* = 0.09, df = 19). (**C)**: HLA-A allele distribution in 41 fASD and 31 female sibs (fSIBS) (*p_c_* = 0.07, df = 12) * (*p* = 0.03, *p_c_* = 0.36; OR: 2.4; 95%CI: 1.1–5.5); § (*p* = 0.006, *p_c_* = 0.07; OR: 0.27; 95%CI: 0.1–0.7. (**D**): HLA-A allele distribution in 137 mASD and 16 male sibs (mSIBS) (*p_c_* = 0.11, df = 13).

The HLA-A, B, C, and DRB1 allelic distributions of 178 ASD children, their 94 SIBS and healthy controls (as reported by Rendine et al. 2012) [23] are shown in Figure 1, Figure 2, Figure 3 and Figure 4, respectively, and in the Supplementary Tables S1–S4. Chi-square comparisons were performed using 2XN contingency tables both between the overall groups (ASD vs. HC and ASD vs. SIBS) and between ASD and SIBS after clustering by sex.

Significant associations of HLA alleles were observed to differ in males and females with ASD compared to their sex-matched siblings and healthy controls, suggesting the existence of sex-related HLA alleles associated with risk and/or protection against ASD, particularly in females.

### 2.1. HLA-A Locus

A comparison of the distribution of HLA-A alleles between children with ASD and HC [23] showed a corrected *p*-value *p_c_* = 0.05 according to the Bonferroni correction, as described in statistical analysis, which is borderline at the significance threshold of *p_c_* < 0.05.

Comparison of the same distribution in ASD vs. SIBS did not evidence any statistical skewing either (*p_c_* = 0.06, df = 13) (Figure 1A, Table S1).

HLA-A allele distribution analyzed according by sex was not statistically different when HC were compared to either female children with ASD (fASD) (*p_c_* = 0.05, df = 19) (Figure 1B) or male children with ASD (mASD) (*p_c_* = 0.09, df = 19) (Figure 1B, Table S1). The comparison between children with ASD and siblings matched by sex did not show any significant difference either (fASD vs. fSIBS: *p_c_* = 0.07, df = 12) (Figure 1C) (mASD vs. mSIBS: *p_c_* = 0.11, df = 13) (Figure 1D). A significantly higher HLA-A*02 frequency was nevertheless observed in fASD (34.1%) compared to fSIBS (17.7%) (*p* = 0.03, *p_c_* = 0.36; OR: 2.4, 95%CI: 1.1–5.5). Conversely, HLA-A*03 was less frequently carried by fASD (8.5%) than by fSIBS (25.8%) (*p* = 0.006, *p_c_* = 0.07; OR: 0.27, 95%CI: 0.1–0.7), though these differences were not statistically significant after the correction for the degrees of freedom (Figure 1C, Table S1).

### 2.2. HLA-B Locus

The comparison of HLA-B allele distribution between children with ASD and HC evidenced the presence of a statistically significant skewing *p_c_* < 0.001 df = 36 (Figure 2A, Table S2). Thus, the HLA-B*14 allele was less frequently carried by overall children with ASD (3.37%) compared to HC (7.46%) (*p* = 0.002, *p_c_* = 0.07 OR: 0.4; 95%CI: 0.2–0.8). This skewing was confirmed when mASD (3.65%) were compared to HC (*p* = 0.01, *p_c_* = 0.36 OR: 0.5; 95%CI: 0.2–0.8), but it did not reach the statistical significance when fASD (2.44%) were compared to HC (*p* = 0.06) (Figure 2B, Table S2).

On the contrary, a higher frequency of HLA-B*38 was observed in overall children with ASD (6.74%) compared to HC (2.46%) (*p* < 0.0001, *p_c_* < 0.001 OR: 2.9; 95%CI: 1.9–4.4); this difference maintained statistical significance also after correction for 36 df (Figure 2A, Table S2). This skewing represented a higher risk factor in fASD (10.98%) (*p* < 0.001, *p_c_* = 0.01 OR: 4.9; 95%CI: 2.2–3.4) than in mASD (5.47%) (*p* = 0.006, *p_c_* = 0.21 OR: 2.3; 95%CI: 1.3 −3.9) after comparison with HC (Figure 2B, Table S2). Finally, the comparison between ASD and overall SIBS (both males and females) did not reveal any statistical difference (*p_c_* = 0.29, df = 29) (Figure 2A, Table S2). None of the female SIBS carried the HLA*B38 allele, and the comparison between fASD and fSIBS showed the presence of a significant difference (*p_f_* = 0.01, *p_c_* = 0.24, df = 24) (Figure 2C, Table S2), whereas no statistical difference was observed for this allele when comparing mASD (5.47%) and mSIBS (3.13%) (*p_f_* = 0.97) (Figure 2D, Table S2).

HLA-B*44 was statistically more frequently carried by children with ASD (10.11%) compared to HC (7.03%) (*p* = 0.03, *p_c_* = 0.72 OR: 1.5; 95%CI: 1.1–2.1) and by their SIBS (12.77%), but this skewing was not statistically significant (*p* = 0.05) (Figure 2A, Table S2). Specifically, HLA-B*44 was more frequently carried by mASD (10.95%) compared to HC (*p* = 0.02, *p_c_* = 0.68 OR: 1.6; 95%CI: 1.1–2.4) (Figure 2B, Table S2). No difference in HLA-B*44 distribution was observed between mASD and mSIBS (12.5%) (Figure 2D, Table S2) or between fASD (7.32%) and fSIBS (12.9%) (Figure 2C, Table S2).

Finally, the results showed that HLA-B*27 was less frequently carried by mASD children (2.19%) than by healthy mSIBS (21.88%) (*p* = 0.0001, *p_c_* = 0.003 OR: 0.08; 95%CI: 0.02–0.3) (Figure 2D, Table S2).

### 2.3. HLA-C Locus

HLA-C distribution was statistically different between HC and children with ASD (*p_c_* = 0.006, df = 13) (Figure 3A, Table S3). In detail, the HLA-C*04 allele was less frequently carried by children with ASD (14.60%) compared to HC (19.48%) (*p* = 0.02, *p_c_* = 0.26 OR: 0.7; 95%CI: 0.5–0.9). Notably, the frequency of the HLA-C*04 allele was lower in both mASD (14.6%) (*p* = 0.04, *p_c_*= 0.52 OR: 0.7; 95%CI: 0.5–1.0) and fASD (14.6%) compared to HC, although in the latter case, differences did not reach statistical significance (*p* = 0.27) (Figure 3B, Table S3).

In contrast, HLA-C*12 was more frequently carried by children with ASD (15.45%) than by HC (10.87%) (*p* = 0.01, *p_c_* = 0.13 OR: 1.5; 95%CI: 1.1–2.0). A higher frequency was observed in fASD (18.29) compared to HC (*p* = 0.05, *p_c_* = 0.65 OR: 1.8; 95%CI: 1.0–3.2), whereas no differences were observed between ASD and their SIBS as a whole. However, after clustering SIBS by sex, a significantly lower frequency of HLA-C*12 was observed in healthy fSIBS (3.23%) compared to fASD (*p_f_* = 0.008, *p_c_* = 0.09 OR: 6.6; 95%CI: 1.6–44.5) (Figure 3C, Table S3).

### 2.4. HLA-DRB1 Locus

The comparison of HLA-DRB1 alleles between ASD and HC revealed a statistical skewing (*p_c_* < 0.001, df = 12) (Figure 4A, Table S4). Specifically, HLA-DRB1*11 was significantly less frequent in children with ASD (21.35%) than in HC (29.17%) (*p* = 0.001, *p_c_* = 0.01 OR: 0.7; 95%CI: 0.5–0.8). This skewing stemmed from the difference between mASD (20.07%) and HC (*p* = 0.001, *p_c_* = 0.01 OR: 0.6; 95%CI: 0.4–0.8), whereas HLA-DRB1*11 frequency was similar in HC and fASD (26.25%) (*p* = 0.5) (Figure 4B, Table S4)

Conversely, HLA-DRB1*07 was statistically more frequent in children with ASD (14.89%) than in HC (10.87%) (*p* = 0.02, *p_c_* = 0.24 OR: 1.4; 95%CI: 1.1–1.9) (Figure 4A, Table S4). However, this difference did not reach statistical significance when HLA-DRB1*07 frequency in mASD (14.60%) and fASD (16.25%) children were compared separately with HC (*p* = 0.06 and *p* = 0.6, respectively) (Figure 4B, Table S4). Finally, no statistically significant differences were observed when comparing the HLA-DRB1 distribution in children with ASD and their SIBS both as a whole (*p_c_* = 0.86, df = 12) (Figure 4A, Table S4) and sex-matched: fASD vs. fSIBS (*p_c_* = 0.95, df = 12) (Figure 4C), mASD vs. mSIBS (*p_c_* = 0.72, df = 12).

### 2.5. Transmission Disequilibrium Test

Finally, we evaluated the possibility of a preferential transmission from parents to children with ASD and their SIBS of the HLA alleles associated with ASD risk and/or protection (Table 1). TDT analysis evidenced that HLA-A*02 was preferentially more transmitted (T = 18) rather than not transmitted (NT = 7) from parents to fASD (*p* = 0.03). Notably, 2 × 2 chi-square analysis showed that HLA-A*02 was also statistically more frequently transmitted than not transmitted to fASD than to mASD (*p_f_* = 0.02, OR: 3.12; 95%CI: 1.2–808). No difference in parental transmission was observed for SIBS.

HLA-A*03 2 × 2 chi-square comparison showed that this allele had a higher frequency of transmission to healthy SIBS (T = 21 vs. NT = 10) than to children with ASD (T = 21 vs. NT = 31) (*p_f_* = 0.02, OR: 0.3; 95%CI: 0.1–0.8); in particular, HLA-A*03 was more frequently transmitted than not transmitted to fSIBS (T = 15 vs. NT = 7) (*p* = 0.08), although not statistically significant, whereas no difference was observed in either mSIBS or ASD children.

TDT analysis showed a higher frequency of HLA-B*38 transmission (T = 21) than non-transmission (NT = 6) in children with ASD (*p* = 0.004), and in particular, it was more frequently transmitted to fASD (T = 8 vs. NT = 1) (*p* = 0.02); HLA-B*38 was also more frequently transmitted to mASD (T = 13 vs. NT = 5) but this did not reach statistical significance. HLA-B*38 was less transmitted to healthy SIBS (T = 1 vs. NT = 6) (*p* = 0.06), and 2 × 2 chi-square analysis showed a preferential transmission of the HLA-B*38 allele to all ASD children (T = 21 vs. NT = 6) compared to their healthy SIBS (T = 1 vs. NT = 6) (*p_f_* = 0.008).

Finally, the HLA haplotypes of ASD children and their siblings were inferred from parental segregation, and frequencies were estimated by direct counting. A significantly higher transmission of the HLA - B*38-C*12 haplotype was observed only in fASD (T = 7 vs. NT = 1) (*p* = 0.03).

## 3. Discussion

The prevalence of ASD diagnosis is lower in females than in males [1], which has been partly explained by the ability of females to mask autistic tendencies, leading to under- or misdiagnosis of ASD [24,25]. In addition, protective factors may reduce the risk in females, and/or a higher burden of risk factors plays a role in this phenomenon [7]. However, despite the known sex differences, genetic studies in ASD have primarily focused on male or mixed-sex samples, limiting the understanding of female-specific genetic contributions [26,27,28]. Given the important role that HLA class I and II genes play in the development of ASD [22], it is plausible to hypothesize that HLA class I: A, B, C, and class II: DRB1 alleles may influence ASD risk and/or sex-associated protection.

Our results indicate that HLA-B*14, C*04, and DRB1*11 alleles are less frequently carried by children with ASD than by their healthy SIBS and healthy controls, suggesting a possible protective role of these alleles towards the development of ASD. Notably, the results also indicated that some HLA alleles were differentially associated with ASD in relation to sex. In particular, HLA-A*02, B*38, and C*12 were found to be more frequently carried by girls with ASD compared to both healthy controls and healthy female siblings. However, they were not differentially distributed in males with ASD compared to their male siblings, suggesting that they may be possible risk factors for ASD, specifically in females.

HLA-A*03, on the other hand, which was carried less frequently by female ASD children than by female healthy SIBS and HC, could be considered a protective factor for female children at risk. Finally, HLA-B*44, which was skewed in boys with ASD, may be an ASD risk factor only for males.

TDT analysis, which allows for a more in-depth assessment of the transmission of individual alleles from heterozygous parents to offspring, confirmed the preferential transmission of the HLA-A*02 and B*38 “risk alleles” by parents to female children with ASD. Conversely, a higher transmission of the HLA-A*03 “protective allele” to healthy daughters was observed. In contrast, no particular differences in parental transmission to male children with and without ASD were found.

Among all the possible *HLA* class I and class II allelic haplotypes, the HLA B*38-C*12 haplotype was more frequently transmitted to female children with ASD alone. This haplotype is part of the ancestral CEH 38.1 haplotype, which maps in a polymorphic frozen beta block region on chromosome 6p21. Notably, an association with an extended haplotype, including HLA-B*38-*C12, has previously been described in Italian children with ASD; no sex-specific association was evaluated at that time [29].

The results reported here would suggest the existence of a different HLA risk pattern for ASD in females than in males, supporting the hypothesis of a sex-related etiology for the development of ASD [7].

Furthermore, the observation that the sex effect in ASD in our case study was exclusively highlighted within HLA class I alleles may be supported by the suggestion that biological sex may influence the association of HLA class I molecules with the T cell receptor V beta chain, thereby influencing T cell selection and expansion which may have implications for disease bias [30].

Furthermore, since all nucleated cells express HLA class I molecules on their surface, we cannot exclude that the interaction between sex-dependent expression of HLA class I molecules and neurodevelopment is not immune-mediated. HLA class I antigens are expressed by somatic brain cells involved in neurodevelopment and play an important role in fundamental functions of the CNS, including neurogenesis, neuronal differentiation and migration, and synaptic plasticity [20].

During the initial establishment of connections within the cerebral MHC-I molecules was suggested to negatively regulate synapse density by controlling AMPA receptor trafficking at the synapse. Modification of MHC-I surface expression also alters the balance between excitation and inhibition of cortical neurons, with serious implications for functional connectivity within the cerebral cortex [31]. Thus, MHC-I molecules act to bidirectionally regulate the initial establishment of connections within the early postnatal cortex, a time point thought to be critical for the developmental defects that cause ASD [32]. On these bases, we may hypothesize that (1) the sex-specific expression of different HLA class I antigens may differentially modulate neural processes in ASD and (2) the effect of MHC-I expression described above may have a different influence in males and females, depending on the different pathophysiology of ASD development according to sex [33]. Indeed, males and females show different cortico-cerebellar connectivity profiles, with females showing cortico-cerebellar hyperconnectivity in contrast to hypoconnectivity in males [34].

Previous studies have yielded controversial results regarding the association of HLA with ASD, mainly due to ethnic and geographical differences but also due to different methodological approaches from GWASs to association studies [7,15,16]. It is important to note that the phenotypic variability of ASD manifestations is hardly taken into account, partly due to the small number of cases analyzed, and no HLA association studies have been performed in relation to sex. Data herein and the above observations suggest that in-depth analyses of associations between HLA expression and ASD should be designed upon dividing patients according to their biological sex, which may also influence their phenotypic manifestation.

## 4. Material and Methods

### 4.1. Patients and Controls

A total group of 178 children with a diagnosis of ASD according to the DSM-5 criteria [1] were enrolled at the IRCCS Mondino Foundation National Neurological Institute of Pavia (Pavia, Italy) and the IRCCS Fondazione Don Gnocchi, Milan (Italy). One hundred thirty-seven of the children were males (mASD) (mean age at diagnosis ± ds) (3.73 ± 1.49), and 41 were females (fASD) (3.75 ± 1.88). All the available healthy SIBS were enrolled as well in the study; this group included 16 brothers (mSIBS) (mean age at enrollment ± ds) (10.25 ± 5.5) and 31 sisters (fSIBS) (11.5 ± 5.9). A total of 277 parents (132 fathers, 145 mothers) of ASD children were finally enrolled to evaluate intra-familial genetic transmission. All the children underwent clinical, psychiatric, neurological, and neuropsychological evaluation, and ASD diagnosis was confirmed by the Autism Diagnostic Observation Schedule 2 (ADOS-2) [35], the Childhood Autism Rating Scale (CARS) [36], and the structured parent’s interview Autism Diagnostic Interview-Revised (ADI-R) [37]. None of the healthy siblings presented ASD symptoms, and their CARS scores were all in the normal range (<30).

Exclusion criteria were a known genetic syndrome and confirmed lesion of the central nervous system and/or the diagnosis of intellectual disability and/or other neurodevelopmental disorders without ASD according to DSM-5 criteria.

The study was designed and conducted according to the Declaration of Helsinki; the research protocol was approved by the Don Gnocchi Foundation Ethical Committee (protocol n. 06_18/05/2016) on 18 May 2016.

### 4.2. HLA Typing

Genomic DNA from ASD children, their siblings, and their parents were isolated from peripheral blood or saliva. DNA from blood was obtained using a standard phenol/chloroform procedure, whereas the ORAgene-DNA (DNA Genotek, Ottawa, ON, Canada) was used for saliva samples.

HLA class I-A, B-C, and class II-DRB1 allele typing was performed using the HISTO TYPE SSP KITS (BAG Diagnostic GmbH, Lich, Germany). HLA-specific PCR amplicons were separated using a 2% agarose gel stained with GelRed^®^ (Biotium, Inc., Fremont, CA, USA) and visualized under UV light. For evaluation, positive and negative reactions were analyzed and interpreted according to the specific evaluation worksheet for each HLA locus.

HLA class I-A, B-C, and class II-DRB1 allele distribution in healthy controls was derived from Rendine et al. 2012 [23]. Subjects were from the Italian Bone Marrow Donor Registry (IBMDR) and included individuals recruited as volunteers: 3571 donors were analyzed for HLA-A, 7591 for HLA-B, 4715 for HLA-C, and 57,345 for HLA-DRB1 [23].

All the HLA alleles were reported at two-digit resolution.

### 4.3. Statistical Analysis

Allelic association analysis was performed to assess HLA genetic distribution between groups. N × 2 contingency tables were used: N = number of alleles detected for each HLA locus, and 2 represents the groups compared. Groups were clustered as Healthy controls (HC), total ASD, subdivided into Male ASD (mASD), and Female ASD (fASD), Total SIBS subdivided into Female SIBS (fSIBS), and Male SIBS (mSIBS). Chi-square analysis was performed, and Bonferroni correction for degrees of freedom (df) was applied to calculate *p* values (*p_c_*); df was calculated (as N-1 alleles), and *p_c_* values < 0.05 were considered statistically significant. Post hoc analyses were performed using 2 × 2 tables to assess the association of individual alleles with ASD. Fisher’s exact test (*p_f_*) for small sample sizes (less than five units per cell) was applied to the 2 × 2 table as appropriate. The risk of association was reported as an Odds Ratio (OR) with a 95% Confidence Interval (CI). All statistical analyses were performed using the IBM SPSS 29.0.1.0 statistical software. The significance threshold was set at *p* < 0.05.

The Transmission Disequilibrium Test (TDT) was used to assess allelic association in children with ASD and their SIBS [38]. The TDT simultaneously measures linkage and association by comparing the transmitted (T) and the non-transmitted (NT) allele from a heterozygous parent to the child. Within each nuclear family, parental chromosomes transmitted to children were assumed to carry a susceptibility allele, whereas non-transmitted chromosomes were assumed to harbor a normal allele. In the absence of linkage disequilibrium, the expected frequency of transmitted and non-transmitted marker alleles is 50% each (1:1 ratio). The allele frequency differences between affected individuals and non-transmitted parental alleles at each locus were compared to the expected 1:1 ratio using the one degree of freedom (1df) chi-square test with [39]. The TDT was performed using the S.A.G.E. (v6.4.2) (Statistical Analysis for Genetic Epidemiology), downloadable at http://darwin.cwru.edu/sage/, accessed on 25 June 2024.

## 5. Conclusions

This is, to our knowledge, the first Italian study on HLA association with ASD in relation to sex in both children with ASD and their healthy siblings, providing the scientific community with a description of the frequency of the distribution of HLA alleles in relation to sex in the above-mentioned groups.

Our findings need to be replicated in a larger population and, if confirmed, may provide a useful tool to aid in the diagnosis of ASD, particularly in females, who tend to be diagnosed later in development than males. This, in turn, could facilitate the establishment of early intervention plans targeting the child at risk of ASD. Early intervention is crucial for the management of neurodevelopmental disorders and for better developmental outcomes. This can lead to a reduced need for therapies (such as psychomotor, speech, and physical therapies) and special needs services later in life.

### Strengths and Limitations

Our study has some critical issues: first, the number of children with ASD and their siblings is limited, particularly the number of females with ASD is limited but this is the consequence of the low ratio of females to males with ASD [1]. Due to the small number of subjects, we preferred to compare the HLA allele distribution at a two-digit resolution, which defines the serologically expressed antigen, at the expense of a higher resolution, which would have been more specific but would have given more fragmented results. Another limitation stems from the fact that the HLA distribution in HC was not clustered in relation to sex [23]. In our defense, however, we overcame this limitation by comparing HLA distributions in ASD children and their siblings clustered by sex and, more thoroughly, by assessing preferential transmission within individual families to children with ASD and their siblings.

Healthy siblings are an important comparison group as they share both environmental and genetic risk factors with their siblings with ASD and are at higher risk for developing ASD than in the general population [40]. Therefore, the ability to discriminate risk and protective factors between children with ASD and their siblings may be an important tool for the early identification of siblings at risk.

## Figures and Tables

**Table 1:** Transmission Disequilibrium Test (TDT) performed in ASD children and their SIBS. *P* values of TDT comparison in each group were reported in column TDT. *P_f_* (Fisher exact text for small number) value from 2 × 2 chi-square analysis comparing the number of transmitted versus not transmitted alleles in ASD vs. SIBS was reported in the chi-square column. Statistically significant differences are reported in bold.

		ASD		TDT	SIBS		TDT	Chi Square	OR	95%CI
		T	NT	p	T	NT	p			
A*02	M	35	43	ns	6	5	ns	fASD vs. mASD *p_f_* = 0.02	3.12	1.2–808
	F	**18**	**7**	**0.03**	9	9	ns			
	all	53	50	ns	15	14	ns			
A*03	M	17	24	ns	6	3	ns			
	F	4	7	ns	15	7	0.08			
	all	**21**	**31**	ns	**21**	**10**		ASD vs. SIBS *p_f_* = 0.02	0.3	0.1–0.8
B*14	M	5	5	ns	0	0	ns			
	F	0	1	ns	3	3	ns			
	all	5	6	ns	3	3	ns			
B*27	M	5	5	ns	0	1	ns			
	F	1	1	ns	1	3	ns			
	all	6	6	ns	1	4	ns			
B*38	M	13	5	ns	1	4	ns	mASD vs. mSIBS *p_f_* = 0.06		
	F	**8**	**1**	**0.02**	**0**	**2**	ns	fASD vs. fSIBS *p_f_* = 0.05		
	all	**21**	**6**	**0.004**	**1**	**6**	0.06	ASD vs. SIBS *p_f_* = 0.006	18.8	2.3–505.6
B*44	M	18	15	ns	4	1	ns			
	F	5	3	ns	8	6	ns			
	all	23	18	ns	12	7	ns			
C*04	M	20	27	ns	5	5	ns			
	F	7	7	ns	7	13	ns			
	all	27	34	ns	12	18	ns			
C*12	M	28	23	ns	2	4	ns			
	F	11	8	ns	1	5	ns			
	all	**39**	**31**	ns	**3**	**9**	ns	ASD vs. SIBS all *p_f_* = 0.05		
DRB1*11	M	30	32	ns	10	5	ns			
	F	13	7	ns	6	11	ns			
	all	43	39	ns	16	16	ns			

## Data Availability

The data presented in this study are available upon request from the corresponding author.

## Supplementary Materials

The following supporting information can be downloaded at: https://www.mdpi.com/article/10.3390/ijms25189879/s1.
