# Supplementary material for: HLA-A, -B, -C and -DRB1 Association with Autism Spectrum Disorder Risk: A Sex-Related Analysis in Italian ASD Children and Their Siblings
Source: Int J Mol Sci. 2024 Sep 12;25(18):9879. doi: 10.3390/ijms25189879 (PMC11431861; doi:10.3390/ijms25189879)
Supplement: Supplementary file 1 [file ijms-25-09879-s001.zip › ijms-3179536-supplementary.pdf]

Table S1: HLA-A allele distribution in 3571 HC (Rendine et al 2012), 178 total ASD and 47 total sibs. Also allelic distribution clustered by sex has been reported in 137 male ASD (mASD). 41 female ASD (fASD). 16 male sibs (mSIBS), 31 female sibs (fSIBS)

HLA-A	HC[a] (N= 7142)	mASD[b] (N=274)	fASD[c] (N=82)	mSIBS[d] (N=32)	fSIBS[e] (N=62)	Total ASD[f] (N=356)	Total SIBS[g] N=94	P value	OR	95%CI
	%	%	%	%	%	%	%			
**A*01**	10.92%	9.49%	8.54%	8.54%	4.84%	9.27%	4.26%			
**A*02**	28.73%	27.01%	**34.15%**	34.15%	**17.74%**	28.65%	19.15%	[ce]p=0.03 p_c=0.36	2.4	1.1-5.5
**A*03**	10.99%	11.31%	**8.54%**	8.54%	**25.81%**	10.67%	24.47%	[ce]p=0.006 p_c=0.07	0.27	0.1-0.7
**A*11**	4.90%	7.66%	1.22%	1.22%	6.45%	6.18%	4.26%			
**A*23(9)**	2.30%	1.46%	6.10%	6.10%	1.61%	2.53%	2.13%			
**A*24(9)**	12.41%	14.23%	12.20%	12.20%	12.90%	13.76%	17.02%			
**A*25(10)**	1.90%	0.73%	0.00%	0.00%	0.00%	0.56%	2.13%			
**A*26(10)**	3.71%	5.84%	6.10%	6.10%	6.45%	5.90%	5.32%			
**A*29(19)**	2.44%	4.01%	4.88%	4.88%	9.68%	4.21%	6.38%			
**A*30(19)**	5.56%	4.74%	4.88%	4.88%	1.615%	4.78%	2.13%			
**A*31(19)**	2.00%	2.55%	1.22%	1.22%	3.23%	2.25%	2.13%			
**A*32(19)**	4.20%	3.65%	6.10%	6.10%	3.23%	4.21%	3.19%			
**A*33(19)**	2.10%	2.92%	1.22%	1.22%	3.23%	2.53%	4.26%			
**A*34(10)**	0.11%	0.00%	0.00%	0.00%	0.00%	0.00%	0.00%			
**A*36**	0.11%	0.00%	0.00%	0.00%	0.00%	0.00%	0.00%			
**A*66(10)**	0.59%	0.00%	0.00%	0.00%	0.00%	0.00%	0.00%			
**A*68(28)**	3.70%	4.38%	4.88%	4.88%	3.23%	4.49%	3.19%			
**A*69(28)**	2.69%	0.00%	0.00%	0.00%	0.00%	0.00%	0.00%			
**A*74(19)**	0.10%	0.00%	0.00%	0.00%	0.00%	0.00%	0.00%			
**A*80**	0.55%	0.00%	0.00%	0.00%	0.00%	0.00%	0.00%			
		[ab] p_c=0.09 df=19	[ac] p_c=0.05 df=19	[bd]p_c=0.11 df 13	[ce]p_c=0.07 df=12	[ag] p_c=0.05 df=19	[fg] p_c=0.06 df=13			

N= number of alleles; %=Allelic frequency; p_c=p corrected for degree of freedom (df)

Statistically significant differences are reported in bold

Table S2: HLA-B allele distribution in 7591 HC (Rendine et al., 2012), 178 total ASD and 47 total sibs. Also allelic distribution clustered by sex has been reported in 137 male ASD (mASD), 41 female ASD (fASD), 16 male sibs (mSIBS), 31 female sibs (fSIBS)

HLA-B	HC[a] (N= 15182)	mASD[b] (N=274)	fASD[c] (N=82)	mSIBS[d] (N=32)	fSIBS[e] (N=62)	Total ASD[f] (N=356)	Total SIBS[g] (N=94)	P value	OR	95%CI
	%	%	%	%	%	%	%			
B*07	3 86%	5.84%	3.66%	6.25%	3.23%	5.34%	4.26%			
B*08	4.27%	6.93%	4.88%	0.00%	4.84%	6.46%	3.19%			
B*13	2.35%	4.74%	1.22%	0.00%	6.45%	3.93%	4.26%			
B*14	**7.46%**	**3.65%**	2.44%	9.38%	4.84%	**3.37%**	6.38%	[af]p=..002 p_c=0.07<br>[ab]p=0.01 p_c=0.36	0.4<br>0.5	0.2-0.8<br>0.2 -0.8
B*18	7.57%	8.03%	10.98%	0.00%	12.90%	8.71%	8.51%			
B*27	1.87%	**2.19%**	2.44%	**21.88%**	0.00%	2.25%	7.45%	[bd]p< 0.001 p_c=0.003	0.08	0.02-0.3
B*35	17.38%	15.33%	14.63%	0.00%	11.29%	15.17%	7.45%			
B*37	0.94%	0.36%	1.22%	3.13%	3.23%	0.56%	3.19%			
B*38	**2.46%**	**5.47%**	**10.98%**	3.13%	**0.00%**	**6.74%**	1.06%	[af]p<0.0001 p_c<0.001<br>[ac]p<0.001 p_c= 0.01<br>[ab]p=0.006 p_c=0.21<br>[ce]p_f=0.01 p_c=0.24	2.9<br>4.9<br>2.4	1.9 -4.4<br>2.2 -3.4<br>1.3 -3.9
B*39	2.29%	1.82%	4.88%	0.00%	1.61%	2.53%	1.06%			
B*40	3.77%	1.46%	1.22%	3.13%	3.23%	1.40%	3.19%			
B*41	1.29%	1.46%	0.00%	0.00%	0.00%	1.12%	0.00%			
B*42	0.09%	0.00%	0.00%	0.00%	0.00%	0.00%	0.00%			
B*44	**7.03%**	**10.95%**	7.32%	12.50%	12.90%	**10.11%**	12.77%	[af]p=0.03 p_c=0.72<br>[ab]p=0.02 p_c=0.70	1.5<br>1.6	1.1-2.1<br>1.1-2.4
B*45	0.36%	0.00%	0.00%	0.00%	0.00%	0.00%	0.00%			
B*46	0.00%	0.00%	1.22%	0.00%	0.00%	0.28%	0.00%			
B*47	0.42%	0.73%	0.00%	0.00%	0.00%	0.56%	0.00%			
B*48	0.04%	0.36%	0.00%	0.00%	0.00%	0.28%	0.00%			
B*49	6.30%	4.01%	3.66%	6.25%	1.61%	3.93%	3.19%			
B*50	2.26%	2.55%	1.22%	3.13%	1.61%	2.25%	2.13%			
B*51	9.17%	9.85%	9.76%	12.50%	14.52%	9.83%	13.83%			

**B*52**	1.43%	1.82%	2.44%	3.13%	1.61%	1.97%	2.13%
**B*53**	0.65%	1.09%	0.00%	0.00%	3.23%	0.84%	2.13%
**B*55 (22)**	1.32%	2.55%	2.44%	0.00%	1.61%	2.53%	1.06%
**B*56(22)**	0.40%	0.36%	0.00%	0.00%	0.00%	0.28%	0.00%
**B*57 (17)**	2.00%	1.82%	3.66%	3.13%	3.23%	2.25%	3.19%
**B*58(17)**	1.30%	1.46%	2.44%	3.13%	0.00%	1.69%	1.06%
**B*59**	0.01%	0.00%	0.00%	0.00%	0.00%	0.00%	0.00%
**B*62**	4.03%	1.09%	3.66%	3.13%	3.23%	1.69%	3.19%
**B*63**	4.22%	2.55%	2.44%	3.13%	1.61%	2.53%	2.13%
**B*70**	2.32%	1.09%	0.00%	3.13%	1.61%	0.84%	2.13%
**B*71**	0.00%	0.00%	1.22%	0.00%	0.00%	0.28%	0.00%
**B*72**	0.00%	0.36%	0.00%	0.00%	0.00%	0.28%	0.00%
**B*73**	0.72%	0.00%	0.00%	0.00%	0.00%	0.00%	0.00%
**B*75**	0.13%	0.00%	0.00%	0.00%	0.00%	0.00%	0.00%
**b78**	0.18%	0.00%	0.00%	0.00%	0.00%	0.00%	0.00%
**Otejrs**	0.13%	0.00%	0.00%	0.00%	0.00%	0.00%	0.00%
		**[ab]p_c<0.001 df=34**	**[ac]p_c<0.001 df=35**	**[bd]p_c=0.005 df=27**	[ce]p_c=0.44 df=24	**[af]p_c<0.001 df=36**	[fg]p_c=0.29 df=29

N= number of alleles; %=Allelic frequency; p_c=p corrected for degree of freedom (df): OR: Odds ratio; 95%IC: interval of confidence

Statistically significant differences are reported in bold

Table S3: HLA-C allele distribution in 4715 HC (Rendine et al., 2012), 178 total ASD and 47 total sibs. Also allelic distribution clustered by sex has been reported in 137 male ASD (mASD), 41 female ASD (fASD), 16 male sibs (mSIBS), 31 female sibs (fSIBS)

HLA-C	HC[a] (N=9430)	mASD[b] (N=274)	fASD[c] (N=82)	mSIBS[d] (N=32)	fSIBS[e] (N=62)	Total ASD[f] (N=356)	Total SIBS[g] (N=94)	P value	OR	95%CI
**C*01**	3.14%	2.19%	2.44%	3.13%	4.84%	2.25%	4.26%			
**C*22**	4.51%	4.74%	2.44%	6.25%	0.00%	4.21%	2.13%			
**C*03**	4.74%	6.20%	9.76%	0.00%	6.45%	7.02%	4.26%			
**C*04**	**19.48%**	**14.60%**	14.63%	18.75%	20.97%	**14.61%**	20.21%	[af]p=0.02 p_c= 0.26<br>[ab]p=0.04 p_c= 0.52<br>[ac]p=0.27	0.7<br>0.7	0.5-0.9<br>0.5-1.0
**C*05**	5.95%	6.20%	6.10%	6.25%	6.45%	6.18%	6.38%			
**C*06**	7.71%	9.49%	7.32%	9.38%	14.52%	8.99%	12.77%			
**C*07**	22.01%	25.18%	24.39%	40.63%	24.19%	25.00%	29.79%			
**C*08**	3.29%	3.65%	2.44%	0.00%	4.84%	3.37%	3.19%			
**C*12**	**10.87%**	14.60%	**18.29%**	12.50%	**3.23%**	**15.45%**	6.38%	[af]p=0.01 p_c=0.13<br>[ac]p=0.05 p_c=0.65<br>[ce]p=0.008 p_c=0.09	1.5<br>1.8<br>6.6	1.1-2.0<br>1.0-3.2<br>1.6-44.5
**C*14**	4.13%	1.09%	0.00%	0.00%	3.23%	0.84%	2.13%			
**C*15**	6.69%	6.20%	8.54%	3.13%	4.84%	6.74%	4.26%			
**C*16**	6.29%	4.74%	3.66%	0.00%	6.45%	4.49%	4.26%			
**C*17**	1.13%	1.09%	0.00%	0.00%	0.00%	0.84%	0.00%			
**C*18**	0.06%	0.00%	0.00%	0.00%	0.00%	0.00%	0.00%			
		[ab]p_c=0.14<br>df=13	[ac]p_c=0.23<br>df=13	[bd]p_c=0.69<br>df=12	[ce]p_c=0.12<br>df=11	**[af]p_c=0.006<br>df=13**	[fg]p_c=0.35<br>df=12			

N= number of alleles; %=Allelic frequency; p_c=p corrected for degree of freedom (df): OR: Odds ratio; 95%IC: interval of confidence

Statistically significant differences are reported in bold

Table S4: HLA-DRB1 allele distribution in 57345 HC (Rendine et al., 2012), 178 total ASD and 47 total sibs. Also allelic distribution clustered by sex has been reported in 137 male ASD (mASD). 41 female ASD (fASD). 16 male sibs (mSIBS) 31 female sibs (fSIBS)

	**HC[a] (N=114690)**	**mASD[b] (N=274)**	**fASD[c] (N=82)**	**mSIBS[d] (N=32)**	**fSIBS[e] (N=62)**	**Total ASD[f] (N=356)**	**Total SIBS[g] (N=94)**	**P value**	**OR**	**95%CI**
**DRB1*01**	8.29%	6.93%	6.10%	0.00%	4.84%	6.74%	3.19%			
**DRB1*04**	8.27%	9.49%	8.75%	12.50%	4.84%	9.27%	7.45%			
**DRB1*07**	10.87%	14.60%	16.25%	15.63%	20.97%	14.89%	19.15%	[af]p=0.02 p_c= 0.24	1.4	1.1-1.9
**DRB1*08**	2.54%	2.55%	5.00%	3.13%	6.45%	3.09%	5.32%			
**DRB1*09**	0.51%	1.09%	2.50%	0.00%	1.61%	1.40%	1.06%			
**DRB1*10**	2.70%	1.46%	1.25%	3.13%	1.61%	1.40%	2.13%			
**DRB1*11**	**29.17%**	**20.07%**	26.25%	31.25%	17.74%	**21.35%**	22.34%	[af]p=0.001 p_c= 0.01<br>[ab]p=0.001 p_c= 0.01	0.7<br>0.6	0.5-0.8<br>0.4-0.8
**DRB1*12**	0.95%	3.28%	1.25%	0.00%	1.61%	2.81%	1.06%			
**DRB1*13**	11.18%	12.04%	11.25%	15.63%	11.29%	11.80%	12.77%			
**DRB1*14**	5.28%	7.30%	3.75%	6.25%	9.68%	6.46%	8.51%			
**DRB1*15**	5.96%	6.93%	10.00%	6.25%	8.06%	7.58%	7.45%			
**DRB1*16**	5.18%	6.93%	5.00%	0.00%	4.84%	6.46%	3.19%			
**DRB1*17**	9.11%	7.30%	5.00%	6.25%	6.45%	6.74%	6.38%			
		[ab]p_c=0.002 12df	[ac]p_c= 0.23 12df	[bd]p_c=0.72 df=12	[ce]p =0.96 df=12	[af]p_c <0.001 12 df	[fg]p_c =0.86 12 df			

N= number of alleles; %=Allelic frequency; p_c=p corrected for degree of freedom (df) : OR: Odds ratio; 95%IC: interval of confidence

Statistically significant differences are reported in bold
